# Effect of trauma onset on personality traits of politically persecuted victims

**DOI:** 10.1186/s12888-016-0853-2

**Published:** 2016-05-17

**Authors:** Krzysztof Rutkowski, Edyta Dembińska, Jolanta Walczewska

**Affiliations:** Department of Psychotherapy, Jagiellonian University Medical College, 31-138 Kraków, Lenartowicza 14 Poland; Department of Internal Medicine and Gerontology, Jagiellonian University Medical College, 31-531 Kraków, Śniadeckich 10 Poland

**Keywords:** Post-traumatic stress disorder, Trauma onset in childhood, Political persecution, Personality assessment, Minnesota Multiphasic Personality Inventory-2

## Abstract

**Background:**

The hypothesis that traumatic experiences in early childhood impact personality formation and psychopathology is well known in psychology and psychiatry, but this is difficult to verify statistically in methodological terms. The aim of this study, conducted with politically persecuted Poles, was to establish the influence of the time when trauma is experienced on the development of psychopathological symptoms.

**Methods:**

The subjects were divided into two groups: those who had experienced trauma before age five (group 1) and those who experienced trauma at an older age (group 2). Subjects in both groups suffered from chronic untreated post-traumatic stress disorder. In order to test the research hypothesis, the Minnesota Multiphasic Personality Inventory-2 profiles of both groups were compared using Student’s t-test, and the Mann–Whitney *U*-test.

**Results:**

Statistically significant between-group differences were found for the F validity scale and the following clinical scales: Hypochondriasis, Depression, Psychopathic deviate, Psychasthenia, Schizophrenia, and Social introversion. All the significantly different scores were higher in the group traumatized in early childhood. People exposed to trauma under age five had profiles similar to those traumatized after age five, but they experienced their symptoms more intensely.

**Conclusions:**

Of clinical significance, higher scores on the psychasthenia, schizophrenia, and social introversion scales, especially on the psychopathic deviate scale, indicated pathology only in the early childhood trauma group. Taken together, these symptoms lead to withdrawal and hindrance of social functioning. This outcome confirms the hypothesis of the influence of various early childhood factors (such as trauma) on personality formation and personality traits in adulthood.

## Background

The hypothesis that traumatic experiences in early childhood impact personality formation and its psychopathology is well known and considered relatively obvious in psychology and psychiatry. In spite of the widespread acknowledgement of its veracity, its impact is extremely difficult to verify statistically in methodological terms. Difficulties arise in evaluating the homogeneous stressors that operated in a patient’s childhood, youth, and adult life without therapeutic input, as well as the clinical symptoms. These difficulties are equally well known, but nevertheless are a major obstacle to identifying with any certitude the causes of childhood psychopathology. The Journal of Personality Disorders and the Canadian Journal of Psychiatry have published articles broadly discussing the difficulties in assessing the impact of childhood on personality formation [1–3].

These problems notwithstanding, scholars have begun to focus in recent years on the long-term consequences of childhood trauma. From the personality psychology perspective, early traumatization could be a cornerstone in the disintegration of self, and the earlier in life the onset of trauma, the more disintegrated the self becomes.

Surprisingly, despite the growing number of studies on the consequences of childhood trauma, no research to date has examined differences in personality profiles in respect to the point of trauma experience: childhood or adulthood. Studies have tended to seek similarities rather than differences, emphasizing that individuals suffering from prolonged traumatization in adulthood, as well as those traumatized in early childhood, are assumed to display personality impairments characterized by avoidant and withdrawal personality traits, an inability to handle frustrating situations manifested as tendencies towards irritability or anger, and reduced tolerance to pain or somatization [4, 5].

The Minnesota Multiphasic Personality Inventory (MMPI-2) questionnaire is widely used in complex psychological post-traumatic stress disorder (PTSD) diagnostics to assess personality traits and degree of psychopathology. By contrast, this tool has been employed relatively seldom to evaluate long-term consequences of childhood trauma. Control scale configurations have been obtained that for many years were thought to typify PTSD, with a raised Infrequency scale (F) and a low Correction scale (K)—an expression of a “cry for help”—and the raised clinical scales Schizophrenia (Sc), Depression (D), and Psychasthenia (Pt), producing the three-point code, 8-7-2 [6, 7]. However, subsequent studies of varying populations of PTSD-diagnosed patients (including civilians) did not confirm the universality of this particular code for all PTSD diagnoses. Various study groups have revealed many different profiles for the two or three most-raised clinical scales [8–12]. To date, in the few studies that have used the MMPI to assess the long-term consequences of childhood trauma, a floating MMPI-2 profile (raised scores on six or more clinical scales) was found among victims of physical or sexual abuse or both [13]. These researchers suggested that this was a long-term correlate of early traumatic experience.

The aim of this study was to establish the influence of the time when trauma is experienced on the development of psychopathological symptoms. The observations and conclusions outlined above justify the research hypothesis that a trauma experienced in the early stages of a child’s development may cause enduring personality disorders, even when it is not experienced with full awareness. These disorders are long-term consequences of the trauma, and the earlier the trauma is suffered, the lesser the awareness of the experience. Not remembering a traumatic event does not offer protection from the development of psychopathologies, and early childhood experiences do influence personality formation.

## Methods

### Participants

The study population of people exposed to trauma consisted of two subgroups: those exposed to trauma before age five (group 1), and those who experienced trauma at a later age (group 2). The age limit dividing the groups was based on the faculty for conscious experience of an external situation, thought capacity permitting foresight of imminent threat, and verbal memory permitting recall and relation of experiences. The age limit of 5 years old also has been cited in psychiatry literature as the age marking the end of the early childhood period when suitable developmental conditions are necessary and the impact of premature separation can take a toll [14]. Well-known researchers in the field of childhood development (Bowlby, Miller and Winnicott) wrote an open letter to the British Medical Journal in 1939, in which they warned against dramatic consequences of evacuating children under the age of 5 years during wartime. They highlighted the risk connected with separation, which could lead to mental disorders. At the same time, attention was focused on the fact that children older than 5 years suffered less than the younger children from the evacuation and the loss of their home [14].

It is thought that psychopathological reactions may take the form of a variety of post-traumatic disorders, such as PTSD, enduring personality change after catastrophic experience, and others mentioned in the 2000 *Diagnostic and Statistical Manual of Mental Disorders* (DSM-IV-TR) [15] and the 1993 *International Statistical Classification of Diseases and Related Health Problems* (ICD-10) [16] diagnostic criteria for research.

Our study group comprised 327 people who had been persecuted for political reasons in Poland in 1939–1968, and who voluntarily reported for diagnosis and outpatient psychiatric and internal medical treatment. The average age was 68 years (med. 68, min. 44, max. 88), group 1: average age was 62.9 years, group 2: average age was 71.2. The statistically significant between-group difference in age (*p* = 0.0000) is directly due to the selection criterion, namely the age of five. Trauma duration was fairly similar in both groups (group 1: 4.9 years; group 2: 4.3 years). Although the differences are statistically significant (*p* = 0.0283), it is highly unlikely that such prolonged traumas are clinically different owing to time of duration. The gender disproportion (group 1: 45.38 % males, 54.62 % females; group 2: 65.48 % males, 34.52 % females; *p* = 0.0003) is due to a distinction between the groups; the children who were persecuted had no association with gender, but the veterans and political prisoners are represented mostly by men.

The group included political prisoners (82 people, mostly traumatized in young adulthood during the imprisonment by Polish authorities in Polish prisons because of anticommunist activity), those deported and incarcerated in the Gulag in Siberia by the Soviet authorities (204 people, traumatized in all ages/any age and sent as adults or children to labour camps in Russia only because of Polish nationality, as an equivalent of ethnic cleansing), former prisoners of Nazi concentration camps (27 people, traumatized in all ages/any age and sent as adults or children to concentration camps only because of Polish nationality, as an equivalent of ethnic cleansing), and war veterans or former prisoners of war (POWs) (67 people, traumatized as young adults during fighting or imprisonment in German POW camps). Some of the subjects had experienced more than one of these traumas. The structure of the study group characterized the various political and war traumas.

The most severe political persecutions of anticommunism were conducted in Poland in 1944–1956 [17]. This included mostly young patriots active in conspiracy who were arrested, tortured during the imprisonment and sent to prisons (or sentenced to death). They were persecuted by Polish communist authorities collaborating with the Red Army. People deported to Siberia were Polish citizens living in the east part of Poland occupied by Russia since 17 September 1939 owing to the Ribbentrop–Molotov pact. The available documents confirm that at least 500 000 people were deported [17]. Up to 200000 people survived and returned to Poland, and were directed to other parts of the country, because after the Yalta Conference eastern part of Poland was incorporated by the Soviet Union. During the deportation they were kept in camps in conditions similar to concentration camps. Prisoners of Nazi concentrations camps were sent by German authorities, and were kept in undernourished conditions in preparation for extermination. About five million Poles were imprisoned, and only 1.5 million survived [18]. War veterans include mostly men traumatized during the fights (wounded) and/or imprisoned in POW camps [18].

The legal status of all examined people was verified by special external national authorities and political background were confirmed in all cases. In general, the time and type of trauma were very similar from a clinical point of view, i.e., all subjects had been exposed to trauma/danger at some point in their life.

None of those participating in the study had ever been treated for post-trauma symptoms, and they all met criteria A1, A2, E, and F for PTSD according to the DSM-IV-TR. Cases that were missing data and did not fulfill the study criteria (organic CNS damage, previously received psychiatric treatment, or experience of any form of trauma other than those listed above (such as a road accident)) were excluded from the study (see the detailed demographic description of both groups in Table 1).

**Table 1:** Demographic description of study group

Variable	Group 1 (*n* = 130)	Group 2 (*n* = 197)	Chi-square *p* value
	M (SD)/N (%)	M (SD)/N (%)	
	Min. – max.	Min. – max.	
Age (years)	62.9 (5.41)	71.2 (5.97)	0.0000
	48–70	44–88	
Trauma duration (months)	59.0 (25.47)	52.1 (30.44)	0.0283
	1–168	1–182	
Gender			0.0003
Female	71 (54.6)	68 (34.5)	
Male	59 (45.4)	129 (65.5)	
Diagnosis as per ICD-10			
PTSD F43.1	81 (63.3)	178 (90.8)	0.0000
Enduring personality change after catastrophic experience F62.0	46 (35.3)	8 (4.1)	
Other	3 (1.4)	10 (5.1)	

The study group was unique on a global scale; it was homogeneous, and the subjects had never received psychiatric treatment. They demonstrated a series of symptoms that could be accurately assessed using psychological tools, and the results possibly linked to the traumas experienced. All study participants gave their informed consent before taking part in the study. The study was approved by the Ethical Review Board of Jagiellonian University Medical College.

### Procedures

The first step was to conduct a psychiatric examination consisting of an in-depth consultation, assessment of the subject’s medical and legal documents, and a description of the subject’s current mental state. Next, a psychological examination was conducted using the MMPI-2 questionnaire. Other psychological tests were performed as indicated, such as the Wechsler Intelligence Scale [19, 20], the Mini Mental State Examination (MMSE) [21], and the Clock Drawing Test [22], to refine the diagnostic procedure and exclude people with cognitive disorders from the study group. Symptoms of dementia or other significant organic CNS damage or exposure to any other trauma (i.e. accident) or any psychiatric treatment were exclusion criteria.

### Personality assessment

The study used the Polish adaptation of the MMPI-2 questionnaire, standardized and validated in 2010 by Brzezińska, Koć-Janucha, and Stańczak [23]. The scores obtained in the MMPI-2 can be verified in various ways [23, 24]. For this study, the following methods were used: number of “cannot say” answers, and the F, Lie (L), and K scales. The premise adopted was that the statistical analyses would disregard the scores of people who had more than 30 “cannot say” answers, an F score over 99 points, and/or a K score over 70 points because such scores on the MMPI-2 in patients with PTSD are extremely difficult to interpret [11, 25]. The study also used the scores on the 10 standard clinical scales. With the exception of the “cannot say” scale scores, all scores on both the clinical and validity scales cited in this study are T-scores, transformed as necessary for compilation of a personality profile.

The validity scales were analyzed for all subjects in order to verify the scores and reject inconsistencies, and to analyze the validity of clinical scales. In total, a group of 117 people was ultimately selected for analysis (group 1: 74 people; group 2: 42 people).

### Statistical analyses

To verify the research hypothesis, a comparison of the MMPI-2 scores obtained in the two study groups was made. The statistical analyses included the Chi-square test, Student’s t-test, and the Mann–Whitney *U*-test.

## Results

### MMPI-2 scales – comparison of the groups

**Fig. 1:**
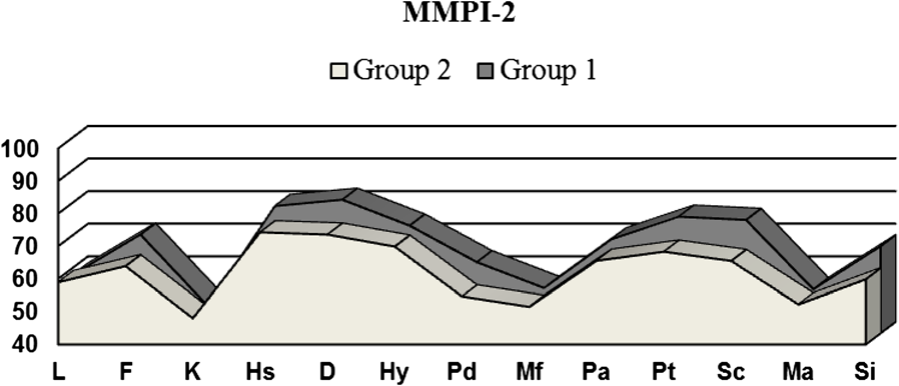
MMPI-2 scales - comparison of the groups. L – Lie, F – Infrequency, K – Correction, Hs –Hypochondrsiasis, D – Depression, Hy – Hysteria, Pd – Psychopathic Deviate, Mf – Masculinity/Fenimity, Pa – Paranoia, Pt – Psychasthenia, Sc – Schizophrenia, Ma – Hypomania, Si – Social Introverion

Statistically significant between-group differences were found for the F validity scale (*p* = 0.0201) and these clinical scales: Depression (D) (*p* = 0.0235), Psychopathic Deviate (Pd) (*p* = 0.0035), Psychasthenia (Pt) (*p* = 0.0079), Schizophrenia (Sc) (*p* = 0.0027), and Social Introversion (Si) (*p* = 0.0022) (see Table 2 and Fig. 1). A significant between-group difference was also found for the Hypochondriasis (Hs) scale using the Mann–Whitney *U*-test (Hs, *p* = 0.0437). All the significantly different scores were higher in group 1, without exception (see Fig. 1).

**Table 2:** MMPI-2 scales - comparison of the groups. T-tests

Variable	M	M	*p* value
	group 1^a^	group 2^b^	(two-tailed)
L	57.84	58.95	0.6170
F	70.11	63.88	0.0201
K	48.24	47.98	0.8775
Hs	78.86	74.26	0.0580
D	80.81	73.55	0.0235
Hy	72.86	69.88	0.2281
Pd	61.64	54.64	0.0035
Mf	53.74	51.48	0.2859
Pa	68.66	65.52	0.2199
Pt	75.59	68.29	0.0079
Sc	74.61	65.48	0.0027
Ma	53.34	52.21	0.4954
Si	66.51	59.83	0.0022

Note. ^a^n = 74, ^b^n = 42, *L* Lie, *F* Infrequency, *K* Correction, *Hs* Hypochondrsiasis, *D* Depression, *Hy* Hysteria, *Pd* Psychopathic Deviate, *Mf* Masculinity/Fenimity, *Pa* Paranoia, *Pt* Psychasthenia, *Sc* Schizophrenia, *Ma* Hypomania, *Si* Social Introverion

### Validity scales F, L, and K

The higher score on the F scale in group 1 confirms again that the level of the score on the F scale reflects the intensity of psychopathological symptoms; it was higher in the group of subjects in which the scores on the clinical scales were also higher. From a clinical perspective it shows that those exposed to trauma in early childhood were characterized by more intense symptoms, they attached greater importance to them, and they experienced a greater sense of discomfort caused by the symptoms. Moreover, subjects who scored higher on the F scale tended to be dissatisfied, had trouble functioning in social situations, and had enduring psychopathology.

No statistically significant differences between the groups were observed in the scores on the L (*p* = 0.6170) and K (*p* = 0.8775) scales, which means that the groups did not differ in their openness to giving answers or in their attitudes towards the study.

### Clinical scales

Scores on the Hs scale differed significantly (Mann–Whitney *U*-test *p* = 0.0437). The mean average score in group 1 was very high, while that in group 2 was among the high scores; at the same time, this was the highest-scoring scale in the MMPI-2 profile of those exposed to trauma when they were older than 5 years. On the D scale, the difference between the groups also was statistically significant (see Table 2). This was the highest scoring scale in the MMPI-2 profile among those exposed to trauma under age five. On the Hysteria scale (Hy) (*p* = 0.2281), the difference between the groups was not statistically significant. The scores in both groups were in the high bracket, indicating the subjects’ poor insight into their mental functioning. On the Pd scale, the difference between the groups was statistically significant. The mean score in group 2 was the only result that differed between the two groups and was within the normal range, while the score in group 1 was outside this range, in the moderately high bracket. On the Masculinity/Femininity scale (Mf) (*p* = 0.4945), the difference between the groups was not statistically significant. The Mf scale was one of two in which both groups scored within normal limits, and moreover, the score was very balanced at close to 50. The averages on the Paranoia scale (Pa) (*p* = 0.2199) were in the high bracket in both groups, though the differences were not statistically significant. The difference between the averages on the Pt scale was statistically significant, and while the group 2 score was much lower, it was still in the band of high scores. The difference between the average scores on the Sc scale was statistically significant; both scores fell within the high bracket, but one was at the bottom end and the other at the top. The averages on the Hypomania scale (Ma) were normal and the difference between them was not statistically significant (*p* = 0.4945). The average scores on the Social Introversion (Si) scale were in the moderately high range for group 2 and high for group 1. This difference was statistically significant. From a clinical perspective, the high scores on the Pd, Pt, Sc, and Si scales were strongly cohesive, particularly within the group of those exposed to stress under age five. Experienced together, these symptoms can lead to withdrawal and impaired social functioning.

## Discussion

These results confirm the hypothesis that the point in the life span when trauma is experienced has a significant impact on the clinical profile of symptoms and on personality traits. While people exposed to trauma under age five had profiles similar to those traumatized after age five, they also demonstrated more intense experience of symptoms, which was visible in nearly all the scales (with the exception of Mf, which is not interpreted in this way). This result was true above all for the validity scale F and the clinical scales D, Pd, Pt, Sc, Si, and Hs. This outcome confirms the hypothesis concerning the influence of various early childhood factors (such as trauma) on personality formation and personality traits in adulthood.

The MMPI-2 profile of the group exposed to trauma before age five included seven raised clinical scales, which are, from the highest: D, Hs, Pt, Sc, Hy, Pa, and Si. The Pd scale also showed moderately high scores. The highest mean scores together produced the three-point code 2-1-7. Among those exposed to trauma at a later age, fewer clinical scales were raised (six), and the configuration was different. The highest scores obtained were, in order, in Hs, D, Hy, Pt, Pa, and Sc, giving the three-point code 1-2-3. MMPI-2 profiles characterized by several raised clinical scales in both groups were in line with current research in both groups, those traumatized after age five and those exposed to trauma in early childhood.

The configuration of the scales in the neurotic triad (1-2-3), dominating in the profiles of people exposed to trauma at a later age, forms a “descending slope” [26], which represents excessive focus on somatic symptoms, occurrence of nonspecific complaints such as nausea, dizziness, sleep disorders, and headaches; feelings of sadness, distress, withdrawal, and other symptoms typical of depressive disorders; poor insight into mental functioning; and a tendency to blame external sources for problems. These people tend not to link their physical complaints with their psychological experiences. The raised Pt scale co-occurring with high values in the neurotic triad adds the components of anxiety and tension to the above psychopathological profile and confirms the chronic course of symptoms [26]. High scores on the D and Pt scales (known as the distress scales) also indicate a high level of suffering and mental discomfort in this group. The “neurotic” scales dominated over the “psychotic” scales, indicating the predominance among this group of somatic complaints, depressed mood, anxiety symptoms, and a lack of satisfaction over symptoms of social isolation and paranoia. Nevertheless, the raised Pa and Sc scales confirm the existence of psychopathological symptoms that affect social functioning. These individuals’ social contacts are characterized by suspicion, a lack of trust, a sense that their environments are insufficiently supportive, and the feeling that they are socially isolated and misunderstood. These symptoms can be associated with trauma and danger for life. The data are similar to scores obtained in other, chiefly civilian groups exposed to trauma in adulthood [27].

The D scale, which showed the highest scores in the MMPI-2 profiles for those exposed to early childhood trauma, points to more profound depression in mood and a depressive style of functioning that dominated the group, including symptoms such as withdrawal, quickness to tire, and incapability of experiencing pleasure. Very high scores (≥75) are linked to deep depression [23], and the average in group 1 was well above that level. Moreover, a seven-point difference in test scores between the groups may be fully reflected in the clinical picture, and at least in the degree of depressive symptoms experienced. This is because the MMPI-2 test does not serve to make clinical diagnoses, but rather to assess the way the patient experiences symptoms.

The Hs scale showed a slightly higher score among those exposed to early childhood trauma, which suggests preoccupation with somatic symptoms across both groups, though in a slightly more intensive form among those traumatized in childhood. This result is partly connected to the actual somatic symptoms occurring in the subjects as long-term consequences of the traumas, chronic stress, and age. Moderately high scores on the Hs scale may occur in people with real chronic somatic complaints [26]. An analysis of internal medicine examinations confirmed statistically significant, more frequent occurrence of somatic illnesses in this group [28, 29].

The scores on the Pt scale indicated more intense symptoms of tension and anxiety in the group exposed to trauma before age five. Aside from the chronic nature of symptoms and the reinforcement of dysfunction, these subjects’ poorer adaptive capabilities linked to the high scores on the Pd, Sc, and Si scales also may affect this score. More severe withdrawal, avoidance of stressful situations, and a low level of activity, typify clinical symptoms in those exposed to early childhood stress. It is telling that in the 1950s, the name given to the set of post-trauma symptoms typical among former concentration camp prisoners—clinically similar to the later named PTSD—was post-camp asthenia [29–31]. The higher scores on the D and Pt scales in this group also point to a significantly higher level of suffering and mental discomfort in the group exposed to trauma when older.

On the Sc scale, the difference between the groups reflected the different ways in which the subjects functioned. Subjects in group 1 were characterized by a strongly schizoid mode of functioning.

The high score on the Hy scale in both groups indicated use of neurotic defense mechanisms such as dissociation, denial, conversion, and displacement.

The high mean on the Pa scale obtained in this study indicates entrenched paranoid personality traits. The assumption can be made that the subjects were characterized by considerable unease, suspicion, expectation of difficulties and obstacles, and a constant sense of danger. These individuals encountered traumas from a hostile environment, without any deliberate action or negligence on their part. They were powerless to influence this environment, their exclusively negative experiences led to the formation of suspicious attitudes and fear of experiencing repeated trauma.

The raised score on the Si scale indicated the presence of social withdrawal and avoidance as enduring personality traits in the group exposed to early childhood stress.

The moderately high score on the Pd scale indicated that people exposed to trauma in early childhood might develop personality disorders. This score is considerably higher than that in the group exposed to trauma at a later age, which points to large clinical differences between the groups and indicates that psychopathic symptoms practically do not reach the level of disorders among people exposed to trauma when they are over the age of five. However, those who suffer early childhood trauma typically will adapt poorly to external situations, be exaggeratedly static in their views and impulsive, and will find it hard to make and sustain social relations. The causes of emotions are usually placed externally. This score is understandable in light of developmental interpretations. Trauma occurring at a time of intensive biological and mental development will have more severe long-term consequences.

The scores on the Ma and Mf scales were within the normal range for both groups. People who are socially well adjusted, open in their relations with others, optimistic, positive, highly active, creative, and enterprising tend to score moderately high on the Ma scale. These styles of functioning are virtually absent from both study groups, as are defense mechanisms connected with reaction formation and use of denial. It would be expedient to consider whether the score is not dependent on the very premise of research conducted many years after the trauma, as it is highly improbable that the subjects could have functioned for such a long period using such defensive mechanisms. Another explanation is connected with the subjects’ ages because with age, scores on this scale decrease in healthy people, and at around 70 years of age persons achieve mean scores of T = 48 [26]. In light of this study, the scores on the Ma scale may be viewed as typical for this age group, and the level of the subjects’ activity as adequate.

The scores on the Mf scale, in turn, indicate that the trauma these subjects experienced did not affect their experience of their own gender. Analysis of the lives of these individuals did not reveal any trauma connected with sexuality (such as rape in childhood), which may be why the scores were within normal range. A question that remains beyond the scope of this study is whether a similar score would have been obtained in a group of people exposed to sexual traumas. No research has explored how the age at which the sexual trauma occurred impacts the MMPI-2 profile and the scores on this scale.

For this study, the researchers sought to describe the enduring consequences of early childhood trauma, and therefore by definition, the subjects had to have experienced the trauma in childhood and the study conducted in adulthood. Moreover, in order for the findings to be as broad and as credible as possible, no other factors are identified in this study group to have come into play between these two events—such as psychiatric treatment—that could distort the ultimate picture of any disorders.

### Limitations

These findings are limited by several factors including the naturalistic study design and difficulties in repeating this study with similar samples, because nowadays, it is almost impossible to find another group with chronic, untreated PTSD as the modern treatment is based on early intervention. The recruitment strategy that included only self-referred subjects seeking treatment could be responsible for the highly symptomatic MMPI-2 profiles. Trauma types in the study group were not completely homogenous, although they were all human-caused traumas related to political persecution. And finally, we did not control for the influence of non-traumatic life stressors that happened later in the life of the participants, although they probably experienced an unfavorable environment after the political trauma isolated them from society and treatment, which are also traumatizing factors. However, these results are rare and provide new evidence of the influence of non-remembered trauma on personality.

## Conclusions

Despite these limitations, the outcomes of the research confirmed in statistical terms that non-remembered trauma experienced in early childhood distorts personality development. From a clinical perspective, the high scores on the Psychopathic Deviate, Psychasthenia, Schizophrenia, and Social Introversion scales are mutually cohesive, and taken together, these symptoms lead to withdrawal and hindrance of social functioning. Particularly telling are the differences on the Psychopathic Deviate scale that indicated pathology only in the early childhood trauma group, while people exposed to trauma after age five showed no signs of enduring functional disorders that might be defined as psychopathic. Moreover, this finding confirmed the positive verification of the research hypothesis and indicates a reaction to the trauma, enduring development of pathological symptoms, and a maladaptive style of functioning in those exposed to early childhood trauma and not remembering it. The results may (at least in part) be extrapolated to other groups of trauma sufferers. At the same time, the results confirmed the impact of unconsciously experienced and non-remembered factors operating in childhood on personality development and the occurrence of pathological symptoms even decades later.

### Ethics

The research has been approved by Ethical Review Board of the Jagiellonian University, no. KBET/110/B/2011.

### Consent to participate

The conset to particiapte was informed and written.

### Consent to publish

Not applicable.

### Availability of data and materials

All statistical data supporting our findings will be shared on individual request.

## Abbreviations

D: depression
F: infrequency
Hs: hypochondriasis
Hy: hysteria
K: correction
L: lie
Ma: hypomania
Mf: masculinity/femininity
MMPI-2: Minnesota Multiphasic Personality Inventory
MMSE: mini mental state examination
Pa: paranoia
Pd: psychopathic deviate
Pt: psychasthenia
Sc: schizophrenia
Si: social introversion
